# Digital Tourism and Wellbeing: Conceptual Framework to Examine Technology Effects of Online Travel Media

**DOI:** 10.3390/ijerph19095639

**Published:** 2022-05-05

**Authors:** Youngjoon Choi, Benjamin Hickerson, Jaewon Lee, Hwabong Lee, Yeongbae Choe

**Affiliations:** 1 Department of International Office Administration, College of Science & Industry Convergence, Ewha Womans University, Seoul 03760, Korea; young.choi@ewha.ac.kr; 2 Department of Community and Therapeutic Recreation, School of Health and Human Sciences, The University of North Carolina, Greensboro, NC 27412, USA; bhickerson@uncg.edu; 3 College of Fine Arts, Hongik University, Seoul 04066, Korea; jaettul@uos.ac.kr; 4 Department of Space Marketing, COEX, Seoul 06164, Korea; 5 Department of Tourism Management, College of Social Sciences, Gachon University, Seongnam-si 13120, Korea

**Keywords:** digital tourism, virtual travel experience, heuristics, affordance, tourism destination image, inclusiveness, well-being

## Abstract

The current pandemic is accelerating the wide-spreading popularity of digital tourism. Given that technology innovation has broadened the horizon of tourist experiences to the realm of virtual environments, this study aims to (re)conceptualize travel experience and develop a theoretical framework to examine media technology effects on virtual travel experience, destination image, and tourists’ well-being. As a conceptual work, this study adopts technological perspectives on online travel media to decompose technology attributes and articulate distinctive effects of technology-centric variables. The proposed framework illustrates five propositions that specify and explain the relationships among technology-centric variables (modality, agency, interactivity, and navigability), three groups of moderators (user-centric, content-centric, and situation-centric variables), virtual travel experience, destination image, and psychological wellbeing. By adopting the variable-centered approach to decompose online travel media, this study provides a new theoretical lens to understand the psychological mechanism of media technology effects in digital tourism. The framework will serve as useful methodological guidelines to conduct experiments to investigate the distinctive effect of a particular affordance or a specific technical feature. The potential benefits of digital tourism to enhance tourists’ wellbeing are discussed by highlighting the environmentally friendly and inclusive aspects.

## 1. Introduction

Online media have developed new ways of producing, distributing, and sharing information. This is especially true in a tourism context, which is often regarded as an information-intense industry [1,2]. Thus, digitalization and technology innovation have profoundly influenced what is considered to be tourist experience [3]. Traditionally, tourist experience was thought to involve escaping from everydayness [4], gazing upon exotic cultures and landscapes [5], seeking authenticity [6], and feeling the heightened moment of being a tourist [7]. This traditional concept of tourist experience inherently requires a physical trip to a destination and the spatial apprehension of immediate surroundings. In this digital age, however, tourists can have an enriched virtual experience without physical movement by exploring a tourist destination with advanced technologies, such as virtual reality and augmented reality [8,9]. Likewise, digital tourism is an emerging phenomenon that can possibly transform the formally established conceptualization of the tourist experience.

In the last two decades, online travel media have been developed to broaden the scope of mobility by collapsing temporal and spatial boundaries in a way that tourists can enjoy a vivid virtual experience of a tourist destination while staying in a fixed location [1]. It is a recent trend to provide meaningful and memorable virtual experiences by incorporating newly advanced technical features (e.g., 360° panoramic images, interactive pictures, and 3D virtual reality) into travel websites/applications [10,11]. Tourists use these travel websites/applications to either enjoy a virtual experience for its own sake [3] or search for information about the destination prior to their physical travel [12]. The recent development of online travel media has opened the age of digital tourism by providing new platforms to enhance the mediated visual perception of a destination. Particularly during the COVID-19 pandemic, many researchers have highlighted its positive benefits to overcome travel constraints because digital tourism is sustainable by enabling ‘carbon emission free’ experiences [13], inclusive to embrace people with mobility disabilities [14], and healthy to enhance psychological wellbeing [15].

However, despite the rapid development of new media technology and its profound impacts, the tourism and marketing literature overlooked to examine what technical aspects of online travel media enrich the mediated travel experience. In addition, the relationship between virtual travel experience and psychological well-being remains understudied. More importantly, there is a lack of theoretical and methodological guidelines in tourism studies to examine distinctive effects of different technological attributes. In the literature on computer-mediated communication (CMC) and human–computer interaction (HCI), researchers have attempted to articulate different technical attributes of online media [16]. No one particular web interface can represent the entirety of online media [17]. Diverse versions of online media have been developed in a complex way of uniquely combining different technical features [18]. Likewise, online travel media utilize various technical features (e.g., motion pictures, user rating systems, interactivity, and 3D virtual reality) to vividly and realistically portray visual images of a tourist destination. Because each technical feature evokes different effects [16,19], it is necessary to differentiate technical features and verify their distinctive psychological effects on tourist experience. In addition, these psychological effects of media technology can be directed toward multiple dimensions of tourist experience, such as the quality of mediated experience and destination images. For example, motion pictures (e.g., a video clip) may evoke a stronger sense of being in a destination but not necessarily create a more positive destination image than still pictures (e.g., a postcard). Hence, there is a need to establish a systematic and comprehensive framework to specify various technological attributes and connect their corresponding effects to the diverse dimensions of tourist experience, which eventually promote sustainable and inclusive tourism and enhance tourists’ wellbeing. 

Drawing on a theoretical and deconstructive approach to investigate online media [16,19], the purpose of this paper is to develop a conceptual framework for conducting scientific studies on examining the impact of media technology on tourist experience. More specifically, this conceptual framework is designed to articulate the distinctive psychological effects of certain technological attributes on (a) virtual travel experience (VTE), (b) the formation of destination images, and (c) tourist’ psychological well-being. Based on dual process models, such as the Elaboration Likelihood Model [20], the Heuristic-Systematic Model [21], and the MAIN (Modality, Agency, Interactivity, and Navigability) model [16], the proposed framework postulates that tourists tend to use visual cues of media technology as heuristics or mental shortcuts in their decision-making process. This work will contribute to the tourism literature and media effect studies by explaining the mechanism underlying how online travel media elicit positive tourist experiences. In addition, the conceptual model will demonstrate the relationships of antecedents, consequences, and moderators to understand technology effects on VTE.

## 2. Three Modes of Touristic Experience

**Figure 1:**
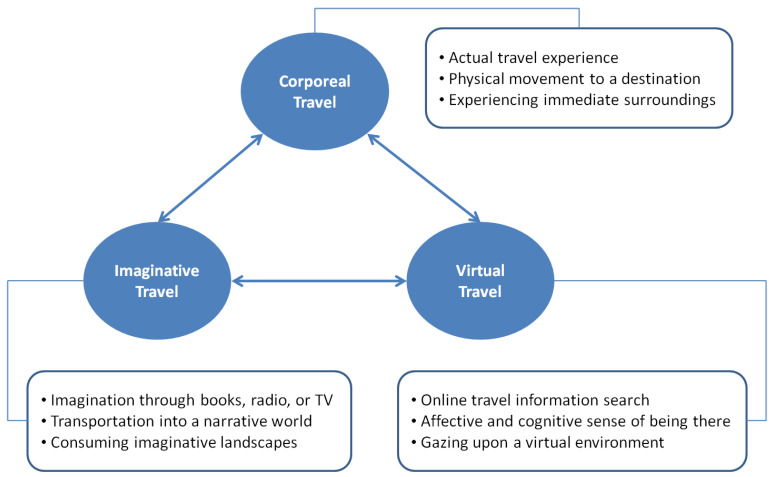
Relations between Corporeal, Imaginative, and Virtual Travel Modes.

According to Urry [5], “there are diverse and interdependent ways of getting ‘there’ (p. 141)”. Based on different ways of consuming a touristic place, three modes of tourist experience can exist: (1) corporeal, (2) imaginative, and (3) virtual travel experience. Corporeal travel is the most intuitive and explicit travel mode, referring to a physical movement to a destination. However, not necessarily involved in physical interactions with the landscapes and infrastructures of a destination, media also provide the channels of conveying a feeling of being in a touristic place. Researchers have suggested people can engage in imaginative travel through books, radio, and TV [22] or virtual travel through online media [1,8,23]. Whereas imaginative travel means the travel mode of consuming imaginative landscapes in a narrative or fantasy world, virtual travel represents the mode of gazing upon realistic landscapes in a virtual environment (Figure 1).

In terms of imaginative travel, media users share meanings and knowledge of the narrative world depicted in novels, TV programs, and movies and create imaginative landscapes of a fantasy world by consuming certain media contents [24]. Researchers in narrative studies found that media users often experience the feeling of being lost in a text by going some distance from their world of origin [25]. According to transportation theory [26], it is possible that individuals can vividly experience the imaginative touristic landscapes by being transported into a narrative world. As “a tripartite formulation” of attention, imagery, and feeling [27], transportation refers to the integrated mental process of being fully absorbed in a story.

On the other hand, with the pervasive phenomenon of experiencing the spatial and cultural landscapes of a destination through online media, VTE refers to the mediated experience of being immersed into tourist destinations. By using virtual tour applications or exploring travel websites, media users appreciate media-induced landscapes of particular touristic places, such as popular national parks or cultural heritage sites. Focused on the feeling of ‘being there’, tourism researchers have recently attempted to conceptualize VTE as ‘telepresence’ or ‘presence’ [10,11]. As one experiential component of virtual reality, telepresence is defined as an individual’s psychological response of a momentary and illusive sense of being in a remote space [28]. 

The recent development of new media technology has increasingly enhanced the quality of both virtual and imaginative travel experiences. Empirical studies have supported that imaginative and virtual travel can be powerful enough to convey enjoyable and memorable experiences, which substantially contribute to tourists’ psychological wellbeing [1,22,23]. However, it is still doubtful that virtual or imaginative travel can replace corporeal travel. Instead of substituting each other, tourism researchers have argued that new media are evoking complex intersections between these different modes of travel [5,22]. In addition, it is a current phenomenon that the three travel modes are rapidly de-differentiated from one another. For example, Reijnders [22] described how tourist experiences of a destination in the modes of corporeal, imaginative, and virtual travel are complicatedly interrelated. Corporeal travel provides tangible places to experience intangible landscapes of the narrative world by being in Lieux d’imagination (places of the imagination). Reijnders [22] proposed that there is a circular process of constructing and consuming the places of the imagination: inspired by physical landscape, artists (e.g., movie makers and novelists) create imagery places and media users engage in the search for the physical references to imagery places. Based on his field works (documentation, participant observation, and in-depth interviews with tourists, tour organizers, and media producers), Reijnders [22] found that the group of tourists inspired by the James Bond films collectively construct a male and romantic gaze to appreciate the exotic landscapes of the movie scenes. 

As online media provides new platforms of creating, distributing, and sharing the high-quality information of a destination, the effects of new media technology on the intersections of corporeal, imaginative, and virtual experience have become more salient than ever. User experience in the process of online travel information search plays an important role to elaborate different modes of tourist experience. Thus, it is important to understand the influence of new media technology on travel information search.

## 3. Media Technology Effects on Virtual Travel Experience

To examine the distinctive effect of diverse media technologies, it is necessary to conduct research based on a variable-centered approach, rather than an object-centered approach [16]. The object-centered approach treats the multiple attributes of a certain medium (e.g., book, TV, and Internet) as a whole. Alternatively, the variable-centered approach considers the unique capabilities within each medium and focuses on detecting the distinctive effect of each of these capabilities [16]. The variable-centered approach is critically important to understand the underlying mechanism of psychological effects because online media have been developed as a heterogeneous form of multimedia. By compiling media effects studies, Sundar [16] adopted the variable-centered approach to articulate different types of affordances of a web interface: each medium has a different set of affordances, which refer to the ontological existence of action possibilities embedded in a given medium. 

The experience of online media users can be dynamically and qualitatively different depending on the unique combination of affordances in a web interface. Previous studies in the tourism literature have found a significant effect of online information search on destination image formation [29] and the existence of different effects between travel agents, tour brochures, and the Internet [30]. However, by adopting the object-centered approach there is a lack of understanding to identify a certain affordance or the underlying mechanism to explain how to produce these effects. By adopting the variable-centered approach, this paper establishes a theoretical understanding to differentiate various forms of online travel media based on technology affordance. In addition, a group of affordances and their corresponding effects on VTE are proposed.

Sundar [16] articulated four types of affordances in the MAIN model: modality, agency, interactivity, and navigability. Modality refers to the mode of communication, such as text, picture, sound, or animated image. Agency means the source of information. Because of multilateral communication patterns in online media, online travel information presents multiple layers of visual cues of sources (e.g., content creator, website, other users, programmer, etc.). Referring to the degree of user control in a web interface, interactivity represents the possibility of taking actions. Navigability is the technological feature determining ways that individuals can explore and move around in virtual space (e.g., linear, left-to-right movement vs. 3D visualization). Because modality, agency, interactivity, and navigability are fundamental technology attributes creating a unique design of web interface, the four groups of affordances in the MAIN model are also applicable to online tourism media. 

Due to a broad spectrum of media technology effects, researchers have argued the effects of a particular technological affordance can be diverse and unique depending on the type of dependent variables [17,19]. For instance, Liu and Shrum [31] proposed that interactivity effects in persuasive communications can be very distinctive based on the locus of consequences, such as learning, self-efficacy, and satisfaction. As the consequences of media technology effects are diverse in a tourism context, VTE can be regarded as a multidimensional concept, and it is important to consider the effects of media technology based on the multiple (affective, cognitive, and conative) dimensions of VTE and destination image. 

**Figure 2:**
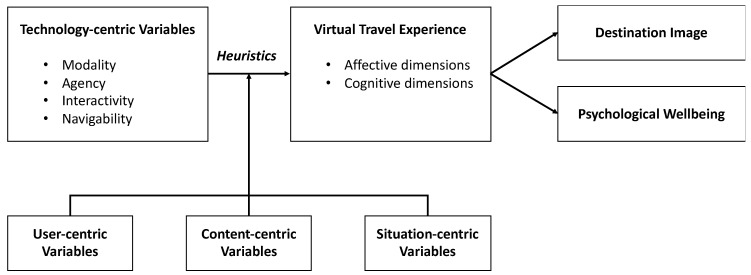
A Framework of Media Technology Effects on Virtual Travel Experience and Destination Image.

In psychology, the elaboration-likelihood model [20] and the heuristic-systematic model [21] propose that humans are considered ‘cognitive misers’ who tend to use heuristics or mental shortcuts in the information evaluation process. In a similar fashion, Sundar [16] contended that visual cues recognized from different affordances elicit unique heuristics (e.g., coolness, bandwagon, and authority), and media users unconsciously or consciously apply these heuristics to evaluate the quality of information or experience in a given web interface. This study proposes a framework describing that affordances of online travel media elicit certain types of heuristics that influence tourists’ evaluation of VTE, which leads to destination image and tourists’ psychological wellbeing (See Figure 2). Moreover, this framework assumes that the direction and magnitude of media technology effects on VTE can be changed by variables not derived from media technology but associated with the media user, the information content, and situational factors. Thus, three groups of variables (i.e., user-, content-, and situation-centric variables) are included as potential moderators in the relationships between antecedents (technology-centric variables) and consequences (VTE). Because this framework is comprehensive by including many groups of variables that should be considered in investigating online travel media effects, the following sections explain how each group of variables influence VTE. In addition, the relationships between antecedents, consequences, and moderators are introduced with a set of five propositions.

### 3.1. Articulating Technology-Centric Variables

In the proposed framework, technology-centric variables are included as antecedents influencing VTE and, consequently, destination image and psychological wellbeing. Technology-centric variables, namely, modality, agency, interactivity, and navigability, would play the most important and meaningful roles in the studies focusing on media technology effects. First, modality indicates affordances determining the mode of communication or the presentation of message (e.g., text, audio, and picture). Unlike traditional media, online travel media can possibly provide a unique set of communication modes. For example, some travel websites may only provide textual information, whereas others may have both textual and pictorial information and even present 3D virtual reality. Some studies have suggested that 3D virtual reality would be the most powerful mode to produce positive user experience [32,33]. However, other studies demonstrated 3D virtual reality could lead to negative outcomes, and there might be an inverted-V effect that the increasing level of modality is no longer positively related to user experience at a certain point [10,34]. For instance, Sundar [34] said that a higher level of modality (a website with pictures and audio files or a website with video clips) is not always better than a medium level of modality (a website with only pictures) in increasing users’ perception about information quality and credibility, possibly because of the amount of required cognitive load. Bogicevic et al. [10] also found similar patterns when comparing the effects of virtual reality and 360 degree images on mental imagery and presence. 

Agency refers to the source of information [16]. For online travel media, the source of information becomes ambiguous [23]. Because online travel media have created a unique distribution channel of information with multilateral communication patterns, online travel information presents the multiple layers of visual cues of sources. For instance, in a situation where a tourist watches a video clip portraying the landscape of a tourist destination on YouTube, there can be a complex set of visual cues implying the sources of information. It can be the creator who uploaded the video clip, the website where it is presented, a user him/herself who chose to watch, or other users who recommended it. Researchers have proposed that these visual cues of sources can elicit profound effects on the perceptions about information [1,23,35,36]. In this regard, even if the visual information about the touristic landscape is identical, it is possible that individuals’ perception of tourist destination image can be changed by source-related visual cues, such as where to view (e.g., specialization of online tourism domain), who approves (e.g., endorsement), and what other people think of the information (e.g., other users’ rating).

Interactivity is the affordances related to the level of control and choices that users have in a web interface. Particularly in the literature on HCI, interactivity usually represents the web interface’s ability of allowing user interaction. By allowing users to interact with either the web interface or other users, interactivity is expressed in various ways (e.g., like button, comment function, chat room, clicking or dragging) on different websites. Many travel websites have been developed to adopt various interactive features to elicit optimal touristic experience and increase tourists’ attitudes and behaviors. It is a recent trend to build travel websites that are interactive as possible, although there is a lack of understanding about the effects of interactive features on VTE. According to Steuer [28], a higher level of interactivity in terms of speed (the rate at which input can be assimilated into the mediated environment), range (the number of action possibilities for action at any given time), and mapping (the ability of a system to map its controls to changes in the mediated environment in a natural and predictable manner) increases a higher sense of telepresence. On the other hand, Sundar [37] posited that a higher level of interactivity does not always induce a more positive user experience. Interactivity features requiring higher levels of cognitive load, data loading time, or buffering may produce negative effects. Similarly, Voorveld et al. [38] asserted that a greater number of interactive features on a website does not necessarily lead to a positive user experience, and it is more important to distinguish particular types of interactive features that contribute to a positive experience. 

Navigability means the affordances determining the level of movement in a web interface. Because of the complex web structure of hyper-linked webpages and multilevel layers, tourists can browse, explore, and navigate in a virtual environment [23]. In particular, by using 3D virtual reality, tourists can move around a virtual environment and gather detailed visual information of the destination [9]. Balakrishnan and Sundar [39] explicated navigability with two components (traversability and guidance) based on the two-dimensional concepts of navigation: (1) travel means physical movement from one location to another location, and (2) way-finding refers to the cognitive process of finding the way to reach the planned location. In terms of travel, traversability is associated with the level of environment constraints and steering control. Related to the concept of way-finding, guidance refers to the scaffolding level to help users find directions. To enhance the perceived reality of movement in a virtual environment, both traversability and guidance may have the power to increase the sense of being in a virtual environment [39].

### 3.2. Effects of Technology-Centric Variables on Virtual Travel Experience

In this study, VTE is defined as the mediated experience of exploring the natural, cultural, and social landscapes of a destination using online travel media. From the perspective of defining authenticity as an individual’s subjective feeling about the unique and genuine tourist experience [40], VTE is also regarded as a real and authentic experience, which is distinct from the experience in a physical environment. In the context of consuming the spatial components of a touristic place in a virtual environment, VTE incorporates multiple aspects of temporality [4], the tourist gaze [41], and the tourist moment [7]. This paper proposes a multidimensionality of VTE, including both affective and cognitive dimensions. 

In the literature on HCI and CMC, the affective dimensions of VTE can be captured with spatial self-location, enjoyment, and arousal. Using online travel media, spatial self-location is defined as the tourist’s feeling of losing the temporal and spatial senses of the immediate surroundings and becoming part of the mediated tourist destination. In their empirical study, Balakrishnan and Sundar [39] contended that certain technological affordances (e.g., navigability features) are positively associated with the level of spatial self-location. Choi et al. [23] supported that higher levels of modality and navigability increased the feeling of being there. Assuming that tourism products inherently involve a hedonic experiential aspect, exploring and navigating touristic places in a virtual environment can generate a certain level of enjoyment [11]. According to the MAIN model, certain technological affordances elicit heuristics (e.g., realism, being-there, flow, contingency, and browsing), which lead to a higher level of enjoyment [16]. Referring to the psychological and physiological state of being awake or reactive to a media stimulus, arousal is related to the continuous pattern of tourists’ heightened moments [7]. Given that certain affordances make online travel information more attractive and realistic, a tourist’s level of arousal can be influenced by the existence or usage of these affordances. 

For the cognitive dimensions of VTE, tourists using online travel media are engaged in the cognitive process of exploring the spatial structure of a virtual environment. According to Urry [41], the tourist experience inherently involves some type of tourist gaze as the process of objectifying and interpreting the place where tourists visit. MacCannell [6] postulated that tourists consistently collect the meanings and structures of a tourist destination by recognizing markers (a piece of information about the space) to organize their touristic experience. As symbols or spatial cues, markers provide tourists the information about the structure of the place. While involved in VTE, tourists also recognize these markers, which deliver the information about the spatial structure of the virtual place. Based on the recognition of markers (or spatial cues), tourists can construct a mental model of the virtual environment [10,23]. Wirth et al. [42] proposed that media users cognitively understand the structure of the virtual environment in their two-step model of spatial presence formation. This model suggests that users first experience spatial presence (affective dimensions) and then construct the mental model of spatial structure in the mediated environment (cognitive dimensions). In this regard, Balakrishnan and Sundar [39] showed certain affordances are positively associated with the level of spatial mental model. Likewise, four technology affordances are closely related to the tourists’ perception about VTE.

**Proposition** **1.** *Technology-centric variables (modality, agency, interactivity, and navigability) may elicit heuristics that influence tourists’ virtual travel experience.*

### 3.3. The Role of Moderators

Researchers have suggested that media technology effects are contingent upon the context of using online media [31,37]. Not associated with the characteristics of media technology, there are potential variables that can change or magnify the effects of technology-centric variables on VTE. Three groups of moderators are included in the proposed framework: user-centric, content-centric, and situation-centric variables. In addition, three propositions are introduced to explain the impact of moderators in the relationship between technology-centric variables and touristic experience. 

User-centric variables are associated with characteristics of the users of online travel media. The proposed framework assumes that these user-centric variables, such as socio-economic status, prior knowledge, immersive tendency, and other potential variables, play the role of moderators in the relationship between technology-centric variables and touristic experience. As younger generations tend to be more active and adoptable for new media technology, Sundar [16] posited the substantial connection between users’ demographic characteristics and the psychological effects of media technology. In addition, particularly for online travel media, prior knowledge about a tourist destination can influence touristic experience. According to Kerstetter and Cho [43], the components of prior knowledge (familiarity and previous experience) influence tourists’ experience in physical destination. Likewise, the effects of technology-centric variables on touristic experience can be more dramatic or marginal depending on the level of tourists’ prior knowledge or technology innovativeness [44]. Witmer and Singer [45] asserted that the tendency of being immersed into a product, a place, or an experience is an individual characteristic in the sense that the distance between-subjects is bigger than the distance within-subjects. For mediated travel, people who tend to be more easily immersed have a stronger sense of virtual experience than others. Thus, the second proposition posits the moderating role of user-centric variables.

**Proposition** **2.** *User-centric variables may magnify or alter the effect of technology-centric variables on touristic experience.*

Content-centric variables refer to the characteristics associated with the attractiveness of a tourist destination. As a central pull factor to increase a tourist’s motivation to visit a certain place, the attractiveness of a tourist destination can reinforce or abate the effects of technology-centric variables. For example, media technology effects would be negligible for some popular tourist destinations where tourists are highly attached. If the message of a popular destination is powerful enough, tourists may not consider heuristics derived from technological affordances in their information search process. In this regard, this framework proposes that the attractiveness of a tourist destination (e.g., reputation and the quality of pictorial image or narrative) has a power to amplify or diminish the effects of media technology on touristic experience.

In addition to reputation, for online travel media, the attractiveness of a tourist destination can be determined by the quality of pictorial information or narrative. In their experimental study, Jun and Holland [46] showed that a higher quality of pictorial information positively influenced individuals’ perception of a tourist destination, regardless of their level of involvement in the decision-making process. According to Green and Brock [26], a well-developed narrative has storytelling power in the context of persuasive communication. Similarly, Busselle and Bilandzic [47] emphasized the importance of storytelling in reducing readers’ counter-argument, elaborating a place based on the narrative and eliciting a stronger sense of immersion. Escalas [48] provided empirical evidence supporting the notion that a narrative message formed with a well-developed storyline has a significantly more positive effect on consumers’ attitude and behavioral intent to purchase than a rhetoric message focused on the explanatory information about a product/service.

**Proposition** **3.** *Content-centric variables may magnify or alter the effect of technology-centric variables on touristic experience.*

Situation-centric variables are associated with the contextual factors of when and how tourists use online travel media. Researchers have suggested there are three different travel stages where tourists are exposed to media: prior to, during, or after a trip. In the process of online travel information search, Tussyadiah and Fesenmaier [3] found that tourists use the travel information from shared video clips on YouTube.com differently based on their stage of travel (i.e., anticipatory, participatory, and reflective use). Tourists may focus on different affordances for each travel stage. The mindfulness of processing the information plays an important role. Gursoy and McCleary [49] proposed two different learning processes associated with media contents based on the channel of exposure: incidental learning and directional learning. When tourists are mindful in directional learning, they are more likely to use heuristics of affordances in information search process. As a leisure activity, media use can be a solitary activity eliciting a sense of presence or a team activity evoking the sense of co-presence. Particularly for online travel media where multiple users can exist in the same virtual environment, the existence of other users may influence tourists’ information search process. Thus, this framework assumes that situation-centric variables of when and how tourists use online travel media moderate the effects of technology-centric variables on VTE.

**Proposition** **4.** *Situation-centric variables may magnify or alter the effect of technology-centric variables on touristic experience.*

## 4. Impact of Virtual Travel Experience on Destination Image and Wellbeing

In the tourism literature, tourist destination image is considered to be one of the most important outcomes of information search/processing. Researchers in psychology and consumer behavior have suggested that image is an abstract, complex, and dynamic concept. For instance, based on the source of information, Gunn [50] differentiated organic images (formed by non-commercial media) and induced images (formed by commercial media). However, considering the multi-labeled sources of online travel media, Li et al. [29] argued that there are blurring distinctions between commercial and non-commercial media in the Internet era, and it is more meaningful to consider the effects of media in terms of users’ level of involvement: baseline (formed by passive information exposure) and enhanced images (formed by active information searching). On the other hand, tourism researchers have distinguished multiple dimensions of destination images. According to Echtner and Ritchie [51], a destination image is a multi-dimensional concept uniquely composed of three continuums: (1) attribute–holistic, (2) functional–psychological, and (3) unique–common characteristics. Similarly, Pike and Ryan [52] posited that a destination image is a tripartite concept formed with affective, cognitive, and conative images. 

Researchers have supported the strong relation between VTE and destination image [9,23,29]. For instance, Gunn [50] regarded media as the main source of constructing both organic and induced images. In addition, Li et al. [29] showed that online information search produces a positive effect on destination image formation. In their mixed-method study (a questionnaire and a short-interview) with 30 undergraduate students to measure the changes in the image of China after a short session of information search (planning travel to China), they found that the affective, cognitive, and conative images of China were significantly and positively improved. Choi et al. [23] found that the search experience of online travel media influenced affective, cognitive, and conative dimensions of the destination image. Recent studies also showed that the experience of using virtual reality positively influenced users’ intention to visit the depicted destination. Particularly during the pandemic, the desire to travel has been suppressed because of imposed cross-border restrictions and increased travel anxiety [15]. VTE can be a substitute of corporate travel and relieve the stress caused by travel constraints. Thus, VTE would increase tourists’ wellbeing.

**Proposition** **5.** *Virtual tourist experience will affect destination image and wellbeing.*

## 5. Discussion

The current pandemic increased the wide-spreading popularity of digital tourism. Considering the environmental and inclusive aspects of digital tourism, the potential benefits of digital tourism to enhance tourists’ wellbeing should be discussed. In this regard, this paper aims to propose a conceptual framework postulating the effects of technology-centric variables (modality, agency, interactivity, and navigability) on VTE, destination image, and tourists’ wellbeing. Based on the variable-centered approach to distinguish diverse technical features of online travel media, the proposed framework is expected to provide meaningful theoretical contributions to the tourism literature. To understand the psychological mechanism of media technology effects, the variable-centered approach is critically important to explain how each affordance is related to positive user experience [17,19]. Although tourism researchers have compared different travel media based on the object-centered approach [30,53], their findings are limited to explain the underlying process of eliciting positive tourist experience. In this regard, the proposed framework will provide a theoretical framework to guide the design of experiments, which are useful to investigate the distinctive effect of a particular affordance or a specific technical feature. Moreover, because the four different affordances are conceptualized as the fundamental units of comprising a particular web interface [16,17,18], this framework is applicable to future online travel media.

In line with dual process models [20,21], this framework proposes that tourists tend to use heuristics derived from recognition or usage of technological affordances in their information search process. By elaborating the linkage between media technology and its psychological responses, this paper will contribute to understanding that heuristics are the key to mediating the effects of technology-centric variables on VTE. In the literature on CMC and HCI, researchers have conceptualized different types of heuristics for each affordance [16,18] and empirically supported that these heuristics influence an individual’s decision-making process [54]. The proposed framework will provide a theoretical foundation for understanding the role of heuristics in the process of online travel information search. For the context of virtual travel, more studies should be conducted to conceptualize and document the sets of heuristics that positively influence touristic experience.

This framework adopts a comprehensive and inclusive approach by containing a volume of different affordances and variables. Because one study cannot examine the effects of the entire set of affordances, it is necessary to conduct a series of different experimental studies to find out the corresponding psychological effects of each affordance. Given that a higher level of technology affordance is not always positively related to user experience [34,37,38,55], it is required to determine a threshold at which a higher level of affordances no longer contributes to positive touristic experience. In addition, as technological affordance may produce dynamic and even conflicting effects depending on dependent variables [16,31], this framework posits that media technology effects should be distinguished based on the multiple dimensions of tourist experience. Although it is not specifically proposed in the framework, it is possible to assume causal relationships between the multiple dimensions of VTE and destination image. For instance, in their empirical study of online shopping, Fiore, Kim, and Lee [56] showed that being in a virtual environment positively influences the level of enjoyment for hedonic shoppers. For future study, it would be meaningful to investigate the relationships among the dimensions of VTE and destination image. Having potential moderators in the proposed framework, this paper suggests considering the effects of technology-centric variables with contextual factors related to the characteristics of a user, the attractiveness of a destination, and the situation of using media.

In terms of developing efficient and immersive web interfaces of online travel media, this study provides useful practical implications. In general, the proposed framework suggests that tourism organizers, destination marketing organizations, and travel marketers should pay more attention to technology-centric variables. For the goal of providing a positive virtual experience, enhancing destination image, and promoting their website, this framework emphasizes the role of technology-centric variables, which have profound psychological effects. Particularly for travel website designers, the proposed framework will provide a systematic framework to test the usefulness of a certain technical feature or compare different versions of web interfaces. Given that a higher level of technology affordance is not always beneficial, the application of emergent technology or the mere inclusion of a number of interactive features is not always the right way to promote positive user attitudes and website loyalty. Instead, this paper proposes that each affordance has different psychological effects on tourist experience. Based on the previous studies with the compelling evidence showing the effects of a particular affordance, web designers should sort out useful technical features that create a cost-efficient and satisfactory web interface.

## 6. Conclusions

Technology innovation has broadened the horizon of tourist experiences to the realm of virtual environments. Given that the current pandemic is accelerating the digital transformation of tourist experiences, this study (re)conceptualized the meaning and scope of travel experience and developed a theoretical framework to examine media technology effects on VTE, destination image, and tourists’ well-being. As a conceptual work, this study adopted technological perspectives on online travel media to decompose technology attributes and articulate distinctive effects of technology-centric variables. This study provides a new theoretical lens to understand the psychological mechanism of media technology effects in digital tourism. The framework will serve as useful methodological guidelines to conduct experiments to investigate the distinctive effect of a particular affordance or a specific technical feature.

Particularly interested in the technical aspect of online travel media, this paper theorizes that affordances can evoke different psychological responses under the condition of processing the same travel information. By including and emphasizing the role of VTE in destination marketing, this paper provides a useful perspective to reconsider the traditionally developed concept of tourist experience and expand its dimensions. Furthermore, as an interdisciplinary approach to synthesize the perspectives of HCI, CMC, psychology, and tourism marketing, the proposed framework is expected to be a theoretical foundation to investigate how digital tourism enhances the quality of VTE, destination image, and tourists’ psychological wellbeing. Based on the proposed framework, future empirical studies should be developed to test the propositions established in this paper to investigate the relationships between antecedents, consequences, and moderators.
